# Collagen: a network for regenerative medicine

**DOI:** 10.1039/c6tb00807k

**Published:** 2016-08-22

**Authors:** K. M. Pawelec, S. M. Best, R. E. Cameron

**Affiliations:** a University of Michigan , 2350 Hayward Ave , Ann Arbor , MI 48109 , USA; b Cambridge Centre for Medical Materials , University of Cambridge , Cambridge , CB3 0FS , UK . Email: rec11@cam.ac.uk

## Abstract

The basic building block of the extra-cellular matrix in native tissue is collagen. As a structural protein, collagen has an inherent biocompatibility making it an ideal material for regenerative medicine. Cellular response, mediated by integrins, is dictated by the structure and chemistry of the collagen fibers. Fiber formation, *via* fibrillogenesis, can be controlled *in vitro* by several factors: pH, ionic strength, and collagen structure. After formation, fibers are stabilized *via* cross-linking. The final bioactivity of collagen scaffolds is a result of both processes. By considering each step of fabrication, scaffolds can be tailored for the specific needs of each tissue, improving their therapeutic potential.

## Abstract 2

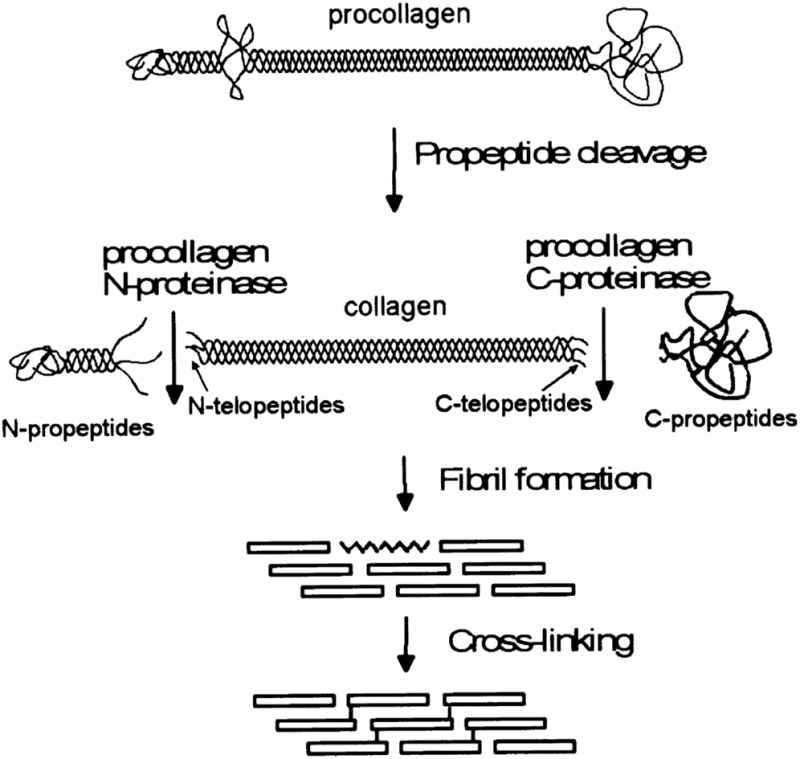
Collagen, as the basic building block of native extracellular matrix, possesses an inherent biocompatibility which makes it ideal for regenerative medicine.

## 1. Introduction

Regenerative medicine focuses on the idea of, not just healing tissue after traumatic injury, but restoring the tissue's native function. The strides made towards this ambitious goal rely on combining advances in medicine with biomedical engineering, a broad field covering diverse topics such as medical imaging, drug delivery and cell therapies. Biomaterials development underpins many of these areas, acting to bridge the gap between therapeutic agents and the body's natural healing response.

Originally biomaterials were inert, designed to elicit no response from the immune system.^1^ With the growth of molecular and cellular biology, a new ideal for medical materials emerged: creating materials which can stimulate native tissue regeneration and restore the original functionality. Constructs of this nature would not only support the overall function of the tissue, but also communicate with the body at the cellular level.^1^ During healing, one means, by which to communicate to cells, is through the diverse cues provided by porous biomaterials scaffolds. The interconnected pores of scaffolds not only support and direct cellular in-growth, but can also be functionalized for drug and growth factor release.^2^ For this versatility, efforts in regenerative medicine often utilize scaffolds.

The materials and methods used to create scaffolds vary greatly. A promising approach has been to utilize natural biological polymers, which already have an innate chemistry to communicate with cells built into the molecule. Of these polymers, collagen is the most widely used, as it is the major structural component of native extra cellular matrix (ECM) in living tissue. Many diverse applications have been found for collagen scaffolds by tailoring their structures, such as osteochondral defects, connective tissues, adipose tissue and mammary glands to name a few.^3–6^ The versatility of collagen scaffolds is due, in part, to the diverse ways in which the structures can be tuned. The pore architecture can be designed to mimic the anisotropic ECM of native tissues, which is important for tissues such as tendon and meniscus.^7–11^ In addition, isotropic scaffolds can by fabricated with pore sizes ranging from 90–300 μm, a parameter affecting cellular in-growth and mechanics, and defined percolation diameters, which affects cellular infiltration.^12–17^ As a further modifier of biological activity, collagen can also be combined with other polymers, such as chitosan or elastin, which influences chemical and mechanical properties.^18,19^

Cellular response to biomaterials is dictated by a combination of mechanical, architectural and chemical cues from the scaffold. Sensing cues from the environment is a complex and dynamic process.^20^ The machinery used by cells, notably a class of trans-membrane receptors known as integrins, can control many functions, including differentiation.^21^ For tissue engineering scaffolds, tailoring biological response is linked to controlling the ligands, or cell signalling moieties, which are presented to cells and react with receptors.

The goal of this review is to examine the formation of collagen networks and how cellular interactions can be tuned *via* these networks. To accomplish this, first the structure of natural collagen is considered along with network formation, known as fibrillogenesis. The physical and chemical properties of collagen fibers are sensitive to many environmental factors, and the properties of collagen, in turn, influence the number and type of ligands which are able to elicit a biological response. Thus, the factors regulating fibrillogenesis can control the migration, phenotype and proliferation of cells in contact with the collagen networks. Once fibers and scaffolds are formed, whether in living tissue or a lab setting, they are invariably modified to enhance mechanical properties, by introducing covalent bonds, or cross-links, between individual fibrils. Cross-links can remove cell adhesion ligands during the process of stabilizing the network. Therefore increasing attention is being paid to finding strategies which stabilize collagen networks while preserving the ligands which make collagen biocompatible. With a deeper understanding of how the chemical environment drives cellular behavior, collagen scaffolds will be one step closer to achieving the goal of regenerative medicine.

## 2. Building a network: collagen fibrillogenesis

Rather than a static environment, cellular function and growth is driven by the ECM and its characteristics – mechanical, chemical, and topographical.^22–24^ Mechanical considerations such as substrate stiffness can act as a cellular signal and cause changes to tissues.^25^ Structural cues, such as pore alignment in scaffolds, can also influence cell behavior, including migration and matrix production.^9,17^ Evidence suggests that cells are sensitive enough to detect and respond to nano-scale changes to the system.^24^ Besides mechanical signals, cells respond to the chemical composition of the ECM.^23^ The chemical environment includes the amount of proteins, ligands, and other network-associated factors which serve as a guide for cell growth. For collagen, the ligands which are able to interact with cells are influenced by the way in which the fibers are formed. The versatility of collagen fibrillogenesis offers researchers a way to fine tune tissue engineering scaffolds for specific tissue environments, especially as the basis of every tissue is a collagen network.

### 2.1 Collagen: the basic building block

Throughout decades of research, it has been determined that collagen is not a single molecule, but a large family of molecules. The defining feature of a collagen is a protein composed of three polypeptide chains incorporating at least one region with a repeating amino acid sequence.^26^ Collagen type I, for example, has a repeating sequence of glycine-X-Y which forms a right-handed triple helix. At least 28 different types of collagen have been found thus far, even excluding the proteins with collagenous regions which have not been called collagen for historical reasons.^26^ Within the collagen superfamily, members are further classified based on their structure and distribution.

The most widespread collagens are the fibrillar collagens, composed primarily of a triple-helical region with a characteristic repeating band where fibers connect, known as D-banding.^26^ A distribution of *D*-spacings exist in the body, ranging between 60–70 nm.^27^ Collagen types I, II, and III all belong to the fibrillar group of collagens and vary in amino acid composition and distribution within the body.^28^ Collagen type I is the most common, constituting the major structural protein for skin, tendon and bone.^28^ Type II, on the other hand, is found almost exclusively within cartilage.^26^

While it is often convenient to classify collagens by the environments where they are most prevalent, many collagens are incorporated in trace amounts throughout the body.^26^ For example, collagen type VI is a constituent of nearly all tissues and has been implicated in tissue integrity.^29^ A growing class of collagens are termed FACITs, or Fibril Associated Collagens with Interrupted Triple helices.^26^ Members of this collagen class associate strongly with collagen fibrils of different types throughout the body, and are believed to have a regulatory role on fiber dimensions and interactions.^30^

Given the wide diversity of collagens and the many functions that the collagens serve, the collagen composition of each tissue is unique. Within many tissues, mature collagen fibers are heterotypic with fibrils from multiple collagen types.^31^ The composition of collagen ECM changes not only with tissue type, but also with age. For example, it has been noted that tendon tissue, which is predominately type I, with traces of types III and V, has varying amounts of type XII and XIV during development, suggesting a regulatory function.^31^ The assembly of heterotypic collagen fibers is a multi-step process which is exemplified by assembly of collagen type I fibers.

### 2.2 Collagen type I fibrillogenesis

Collagen type I fiber formation, termed fibrillogenesis, is a multi-step process driven by the increase in entropy associated with increasing molecular disorder at the water–protein interface.^26^ Within living tissue, it is known that cells play an active role in the deposition of a collagen matrix, but the exact nature of the cellular contribution to the orientation and properties of the matrix remain an area of active investigation. Fibrillogenesis also occurs *in vitro*, although, in general, the collagen networks formed tend to be composed of fibers in a random orientation, rather than the highly aligned bundles, which are normally observed in tissues. The structure of the collagen is important, as this determines the signals, such as adhesion ligands, available for cellular signalling. Thus, the ways in which structure can be modified might be used to direct cell response.

#### Fibrillogenesis in tissues

**Fig. 1:**
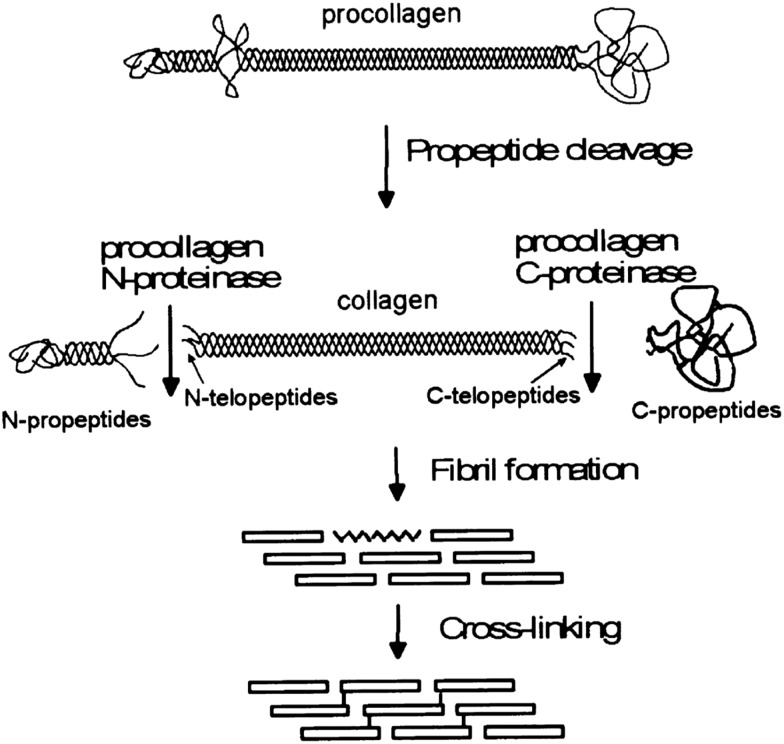
A schematic of the processing stages by which collagen fibers are formed *in vivo*, emphasizing the modifications which occur before fibrils form. Reprinted from ref. 34, copyright 2001, with permission from Elsevier.

The process of fiber formation begins with the translation of the collagen protein in the endoplasmic reticulum. After translation, the peptide is post translationally modified in several ways, including glycosylation, modification of residues, and di-sulphide bonding.^26^ Each collagen molecule contains extra peptide sequences at both ends of the protein which are believed to play a role in the initial formation of a triple helix.^32^ Once three peptides form a triple helix, the molecule is called procollagen.^33^ The end segments are subsequently removed, and the molecule, often called tropocollagen, is stabilized by covalent bonding along the chain and can participate in fiber formation, Fig. 1.^26,34^

Maturation of fibers occurs in three stages: initial formation of fibrils, end to end linear growth, and finally, lateral growth.^31^ All three of these stages occur in the extracellular space, in close association with cells, although there is still debate over the relative importance of cellular ordering *versus* liquid crystal interactions.^26,31,35^ Unlike *in vitro* reactions, which proceed spontaneously, nucleation of collagen fibers *in vivo* is dependent on other molecules, such as fibronectin and collagen type V.^36^ The final stage of assembly, lateral growth of fibers, is only observed in mature tissues, and is highly regulated to ensure that the fiber diameter and orientation are correct.^31^ Molecules such as small leucine rich proteins have been shown to associate closely with collagen fibers to help regulate fiber diameter.^37^

#### Fibrillogenesis *in vitro*

Due in part to the method of harvest, the collagen used in tissue engineering can be extremely diverse. Collagen is often harvested from tissues based on its solubility. The solubility, in turn, depends heavily on the amount of cross-linking present within the collagen fibers, and can also vary between different tissues.^28,38^ Procollagen and immature fibrils are easily solubilized, even in neutral salt solutions, and can be used to form tissue engineering gels.^28^ Further processing of the collagen molecule, during isolation, can remove areas important for normal D-banding, such as the telomere region, leading to many alternative packing arrangements.^39^ The fraction of collagen which is insoluble is held together with mature cross-links, and is thus more resistant to proteases than other forms of collagen.^28^ The number of cross-links which remain in the collagen molecule can also affect the time to form a stable network. More cross-links shorten the time for self-assembly.^40^

It must be noted that a key feature in collagen networks is the characteristic D-banding formed as the individual fibers arrange themselves, revealing an ordered packing along the fiber length. This marks a key difference between collagen and gelatin networks, both of which are used in regenerative medicine. Even though the amino acid sequence is the same, gelatins, or denatured collagen chains, gel by undergoing a conformational change from a random coil to an ordered helix.^41,42^ Within gelatin gels, only very short segments of triple helices form, whose length is dependent on gelling conditions.^43^ With gelatin, the gel is never in an equilibrium state, but is modified over time, increasing the number of helices, but never reaching the state of collagen with D-banding characteristics.

**Fig. 2:**
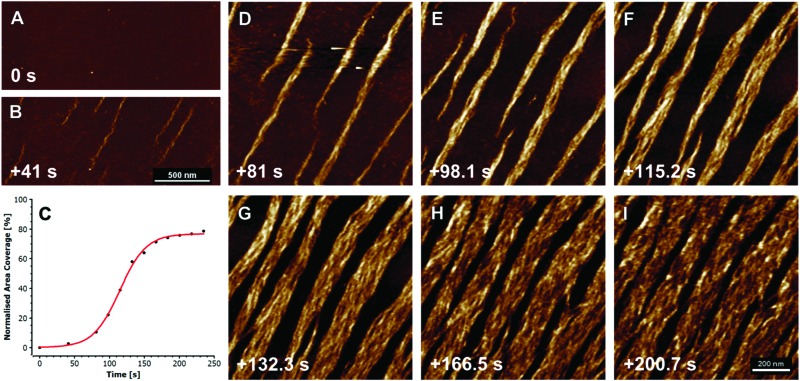
Fibrillogenesis of type I collagen at pH 7.4, at a concentration of 30 mg ml^–1^, imaged with fast atomic force microscopy (AFM). The initial moments are recorded in (A), and subsequent timings in (B and D–I) are measured from this point. First oligomeric intermediates, without D-banding are formed (B), followed by subsequent fiber formation and lateral growth (D–I). The plot in (C) shows the growth of the collagen fibers, as a measure of percent coverage from the images, demonstrating the growth kinetics. X-scan size in (A and B) is 2 mm, and (D–I) is 1 mm; *Z*-height for all images is 3 nm. Reprinted from ref. 47, copyright 2015, with permission from Elsevier.

The formation of collagen fibers *in vitro*, is roughly broken into two phases: a lag and growth phase. During the lag phase of fibrillogenesis, collagen fibrils in solution must associate to form the nucleus of a triple helix structure.^44^ Nucleation proceeds in a step wise manner, starting with the formation of dimers and trimers of collagen fibrils, which then rearrange to form triple helices. However, at high collagen concentrations, collagen molecules can first aggregate, in an equilibrium state with monomers, leading to an elevated local concentration of monomers, and thus assisting the initial nuclei formation.^45^ After a critical number of nuclei are reached, fibrils begin to grow laterally, eventually yielding fibers with the characteristic D-banding seen in natural tissues, Fig. 2. The process is often monitored *via* turbidity measurements, although other methods, such as rapid speed scanning atomic force microscopy (AFM) and confocal reflectance microscopy are being explored.^46,47^

Helix formation relies heavily on electrostatic interactions along the collagen fibrils, and thus, it is very sensitive to environmental factors which can alter the charge along the molecule: pH or ionic strength of the buffer, and the structure of the collagen molecule itself. At pH values far away from the isoelectric point of collagen molecules, fibrillogenesis will be slow or incomplete resulting in a loss of D-banding. In some cases, no fibrils will form unless additional ions or charge altering molecules, are present to force fibril formation by shifting collagen's isoelectric point.^48,49^ The effect of pH can manifest itself as a change in collagen properties, such as swelling, and alteration of the D-banding.^50^ In systems with a dynamic pH, the rate of pH change during fibril formation can affect the size and order of fibrils formed. With higher rates of pH change, the molecules spend shorter periods around the isoelectric point, reducing lateral growth in the fibers.^51^ When the rate is reduced, the lateral growth increases, but some of the D-banding is lost, resulting in a more heterogeneous population of fibrils.^51^

In conjunction with pH, the ionic strength of the buffer solution has a profound effect on fibrillogenesis, generally delaying the onset of nucleation.^44^ Any ions in solution alter the isoelectric point of collagen and can bind to charged groups along the chain, effectively keeping amino acids from participating in the reaction.^52^ Salts also can stabilize the collagen triple helices by screening amino acids or changing how fibrils interact with the water.^48,52^ The type of ions in the buffer also play a profound role in determining the assembly of collagen, through changes in the isoelectric point. For example, with an increase from 0.1 mM to 10 mM KCl, the isoelectric point of pepsin-solubilized collagen type I shifted 7.5 to 5.3, but reached above 9 with the same amount of CaCl_2_.^52^ Complexity is added when multiple ions are present in the environment. For example, in phosphate buffers, chloride ions aid fibrillogenesis of acetic acid soluble collagen much better than in water alone.^49^ In addition, multivalent ions appear to increase the banding formation through increased charge screening along the collagen fibril.^48^ The environment for fibrillogenesis *in vivo* is filled with ions and other molecules. Besides their effect on the stability of collagen fiber formation, they can act as molecular crowders to affect many biological processes. *In vitro*, crowders have been found to shorten the nucleation time and increase growth rates of fibers, by reducing the effective space collagen can occupy, making interactions more likely.^53^

Not just salts, but large molecules can also affect fibrillogenesis. Glycosaminoglycans (GAGs) and small leucine rich proteins (SLRPs) are common in heterotypic collagen molecules and are known to play an important role in the regulation of fiber size *in vivo*.^37,39^ These molecules can be incorporated into growing fibrils during *in vitro* fibrillogenesis, and their properties influence the final structure.^54^ In mixtures of collagen and hyaluronic acid, it was found that the sulphation state of the GAG altered the isoelectric point of the final gels formed, and higher sulphation decreased the fibril size.^54^ Silicates can also can interact with collagen fibrils to control nucleation. When silica nanoparticles were negatively charged, positively charged collagen fibrils associated with the particles, and formed fibers as the pH was lowered.^55^

The amino acid sequence of the collagen molecule also impacts fibrillogenesis in various ways. Packing of the collagen fibrils relies on changes in amino acid conformation along the chain.^56^ A macroscopic effect of the change in structure is readily seen in the different assembly rates of collagen types. Collagen type III and II form fibrils faster than type I, due to higher molecular mass of type I and fewer intermolecular interactions.^57^ The source of collagen also impacts the amino acid structure and the biological signals inherent to the fiber. In fact, the most variable regions between mammalian collagen chains are the sequences of peptides near protein binding sites, which might modify specific cell response.^58^ In addition to mammalian sources, marine collagen is an attractive alternative, as it is cheaper and readily available.^59^ However, collagen from marine sources has very different properties than mammalian collagen, such as lower melting temperatures, due to higher content of glutamic acid and alanine rather than proline.^59^ Bacterial and recombinant collagen proteins are other alternatives to mammalian collagen, containing well-defined amino acid structures which can be modified with additional cell signalling sequences.^60^ Despite the advantages, bacterial collagen has a very low tendency to form fibrils, something which is believed to be caused by a lack of hydroxyproline residues.^60^ While infinitely variable, recombinant proteins are generally limited in size, and thus are closer in nature to gelatins rather than native collagen fibrils. As such, they cannot recapitulate the long triple helices of collagen molecules, and thus any cell adhesive ligands may have an altered conformation in comparison with those found on collagen fibers.^61^

### 2.3 Mature collagen fibers

The formation of fibers is only the first stage in the formation of a collagen structure within the ECM. Several natural processes can occur to the fibers over time, most notably cross-linking. Other modifications to the collagen fiber are typical of disease states. For example, the citrullination of arginine, which forms part of cell binding motifs, occurs frequently in rheumatoid arthritis.^62^ Even natural cross-linking of the collagen fibers can play both a beneficial and harmful role, and is divided into enzymatic and non-enzymatic reactions.

Enzymatic cross-links contribute to the overall biological function by stabilizing the fibers and improving mechanical properties. The cross-links are formed *via* lysyl oxidases (LOX), copper dependent enzymes, which act on the telopeptide region of the molecule.^63^ The enzyme oxidizes lysine residues, recognizing very specific ligands, generally in the telopeptide region of the molecule.^63^ Without LOX, the mechanical development of ECM is arrested, and fibers have irregular size and spacing.^64^ For collagen structures formed *in vitro*, the work of LOX is often taken over *via* chemical or physical cross-linkers.

After the introduction of enzymatic cross-linkers, non-enzymatic reactions take place randomly throughout the helices, accumulating with age. Of the non-enzymatic reactions, glycosylation of the collagen fibers, at arginine and lysine pairs, is the most common and leads to advanced end products (AGEs) which can stiffen the matrix.^65,66^ The AGEs which accumulate often modify the biological behavior of the collagen fibers by interrupting cell and protein binding sequences.^65^ It has been shown that networks formed *in vitro* from collagen incorporating high amounts of AGEs have a higher viscosity and faster gelation than collagen from younger sources, and show a decrease in the proliferation rate of stromal cells.^66^

## 3. Cell recognition of collagen

**Fig. 3:**
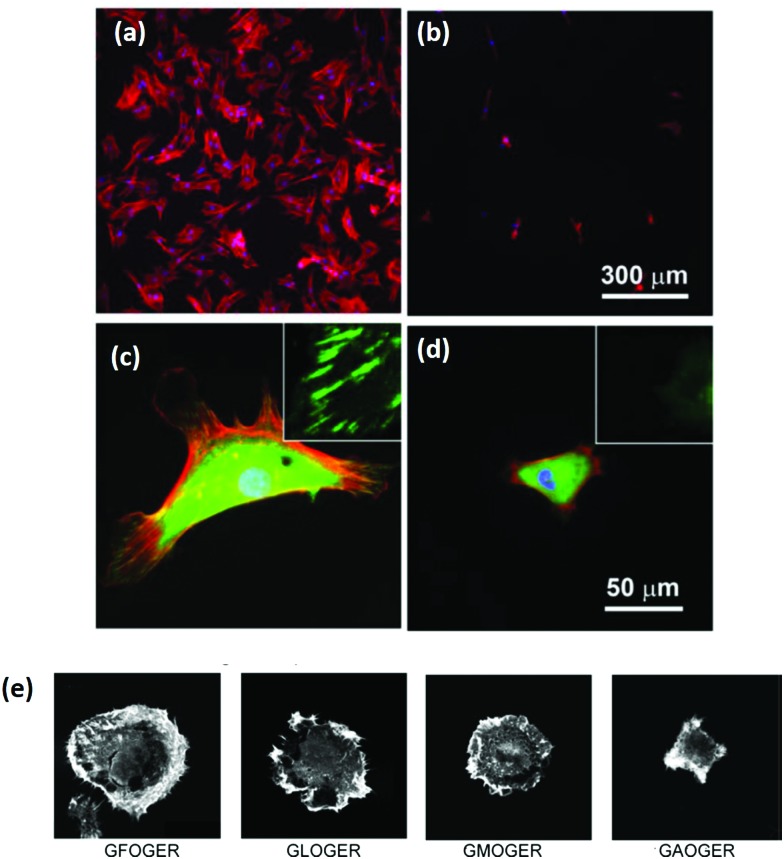
Ligand density of the substrate and ligand affinity affects cellular adhesion and spreading. (a–d) Hydrogels were patterned with RGD either (a and c) 49 nm apart or (b and d) 135 nm apart. After 24 hours, mesenchymal stem cells were stained for cytoskeletal proteins: vinculins (green), F-actins (red), and nuclei (blue). Micrographs were taken at (a and b) low magnification and (c and d) high magnification (insets show close up of vinculin at the cell periphery). Adapted with permission from ref. 68. Copyright 2015 American Chemical Society. (e) The amino acid sequence of the ligand in collagen type I has a profound effect on the affinity, shown in the altered spreading of HT1080 cells, visualized *via* phalloidin staining. Adapted from ref. 69, with permission.

As a key structural component *in vivo*, collagen has an innate biocompatibility, making it attractive for regenerative medicine efforts. Collagen provides many cues to direct cellular behavior. Adhesive ligands, which are naturally occurring in collagen, are powerful regulators of cell response. The importance of adhesion ligands cannot be underestimated. Bone marrow stromal cells require the presence of adhesion ligands to bind to the substrate, as shown in alginate systems, which lack the inherent cues of natural polymers.^67^ In addition, the control of the ligands presented to cells has a profound effect on biological responses, such as cell spreading or stem cell differentiation, Fig. 3.^68,69^ It is the wide range of signals, each of which can be controlled and fine-tuned to some extent, which make collagen a promising tool for controlling tissue regeneration.^69^

Collagen can interact with a variety of cell trans-membrane receptors, including integrins and discoidin domain receptors (DDRs).^70^ One of the most studied class of receptors are the integrins, which link ECM signals to intracellular events, and are often associated with the actin cytoskeleton.^71^ Functional integrins are composed of two subunits: alpha and beta. The alpha subunit recognizes the ligand in the ECM, while the beta subunit generally sets cellular events in motion.^72^ A range of alpha and beta subunits exist, which recognize a variety of ligands, and the downstream effects are often determined by the particular combination of subunits which are activated.^73^ Both the cell type and the developmental stage of the cells influence the integrin subunits which are present at the cell surface.^72^

The cellular processes regulated by integrins are diverse and key to cellular survival, including proliferation, migration and interaction with growth factors.^73,74^ Thus the regulation of integrin binding is extremely important. Regulation can occur by affinities between the ligand and receptor, integrin clustering, and intracellular movement of the integrin receptors.^75^ Mechanical forces also regulate ligand affinities, by stretching areas of the integrin and transitioning manually from closed to open state *via* mechanical means.^76^ Mechanics, and the mechanism of mechanosensing is a complex set of interactions, and has been reviewed elsewhere.^20^ Even shifting from two-dimensional to three-dimensional culture systems can affect the expression of alpha subunits *in vitro*.^74^ Regardless of the type of signal, the downstream effects of integrin binding are mediated by a host of other molecules and proteins, known collectively as the adhesome.^20^

The environment around the cells contains a large number of cues which can be interpreted by cells and plays a role in integrin expression. The ligands which integrins recognize are conserved amino acid sequences found in many components within the ECM.^72^ One of the most well known of the integrin ligands is the RGD sequence, found in proteins such as fibronectin and fibrinogen. In addition to those molecules that use RGD as their primary mediator of cell response, many proteins in the ECM contain RGD sequences which only become accessible after processing or degradation.^72^ Collagen is an example of this type of protein, which only reveals an RGD sequence following denaturation to gelatin. Thus, interactions with native collagen fibers are not through RGD. Instead collagen recognition operates *via* another binding site: GFOGER, which is dependent on collagen's helical structure.^77^ Minor alterations of this sequence also act as ligands, but GFOGER has the highest binding affinity, leading to the greatest attachment, Fig. 3.^68,78,79^ Binding to GFOGER is most commonly through the α_1_β_1_ and α_2_β_1_ integrins, while α_5_, α_V_, and α_8_ subunits recognize the RGD sequence.^72^

Despite the positive effects of incorporating cell ligands into biomaterials, the interaction of the ECM and the environment can also have negative consequences. For example, in a cancerous environment, the ECM undergoes changes, uncovering some of the “hidden” ligands along the collagen fibers. This is especially illustrated by the α_11_ integrin, which plays a role in metastasis in cancer. Due to the stiffening of the matrix, collagen reveals a ligand for α_11_, which is mediated *via* discoidin domain receptors, and leads to cancer activation and metastasis.^80^ For a more detailed treatment of cellular interactions with the ECM in tumor environments, readers are directed to the recent review by Multhaupt *et al.* ^70^

The complexity of ligand interactions increases due to the role of ligand conformation. Surfaces which present RGD in a cyclic conformation trigger different integrin expression than surfaces with linear RGD.^81^ Altering the pattern of integrin activation can affect the composition of focal adhesions at the surface, changing the force generated by the cell, which can ultimately influence complex processes like myogenesis.^81^ Thus, ligand conformation may be an important aspect to consider when designing adhesive peptides or transitioning between collagen and gelatin substrates.

In addition to amino acid sequence, the clustering of integrins has a large impact on the downstream cellular effects, increasing signalling effectiveness, independent of the global density of ligand present, Fig. 3.^68^ However, in a study with α_V_β_3_ integrin binding, it was found that above a certain threshold, integrins lose the ability to bind their ligands due to overcrowding.^82^ Adding to the complexity, cellular processes respond differently to ligands. It was demonstrated that adhesion ligands on surfaces can up-regulate initial attachment but down-regulate later proliferation, migration and matrix production.^83^ Studies with vascular smooth muscle cells show an optimum between 2.8 and 7 μmol ml^–1^ adhesive peptide.^83^ Heterotypic collagen fibers, which incorporate a variety of proteins containing different ligands, can therefore deliver highly specific signals through the type and spacing of signalling ligands. Ligand density acts independently of matrix stiffness to determine the maximum forces generated by cells during adhesion.^84^ The way in which the ligands are anchored in the ECM can also modulate cell expression. A 2.5 fold difference in collagen anchoring density was shown to determine stem cell fate.^85^ This highlights how crucial it is to consider the way in which biomaterials present cellular adhesion ligands to cells. Harnessing the potential of this complex code will be an important step to advance regenerative medicine.

## 4. Harnessing collagen's potential

Despite the ubiquitous nature of collagens *in vivo*, harnessing their potential as biomaterials is an on-going process. The use of collagen as a biomaterial takes many forms, most commonly as gels and scaffolds. Gels are composed of a collagen fiber network whose characteristics are dependent on the fibrillogenesis conditions. Scaffolds incorporate a level of organization at a larger length scale than gels, and thus offer another layer of control. Not only can the fibril structure be tuned, but also the scaffold architecture can be manipulated to form either aligned or isotropic structures of varying pore size.^12^ The many steps to form mature collagen fibers give researchers many stages to tailor the structure and chemistry of collagen materials to meet their particular needs. These can be roughly divided into the stage of initial network formation and network stabilization *via* cross-linking.

### 4.1 Tailoring the collagen network

The sensitivity of collagen fibrillogenesis to environmental conditions make it a natural way to tailor the properties of the collagen network. Given the wide range of diseases which are associated with changes in the structure or chemistry of collagen, it is evident that biological activity is extremely sensitive to alterations which take place during the events of fibrillogenesis.^86^ Indeed, several studies have shown that structural changes to the collagen fiber alter the biological activity. In gels, this is often accomplished through mixtures of collagen with other molecules.

Creating mixtures of collagen and other polymers, such as GAGs, has led to a wide range of biomaterials with differing biological properties. In mixtures of collagen and hyaluronic acid, it was found that additions of sulphate groups to the hyaluronic acid led to a decrease in fiber size after fibrillogenesis.^54^ The extra sulphation also correlated to a decrease in osteoclastogenesis.^54^ However, organic materials are not the only molecules which can be incorporated into growing collagen fibers. Addition of oxidized carbon nanotubes alter the physical characteristics of collagen, such as the D-banding.^87^ With the incorporation of nanotubes, the D-banding increased from 67 to 70 nm, and the stiffness of the fibers increased as well. The conformational change was shown to drive increased neuronal differentiation of human decidua parietalis placental stem cells *in vitro*, compared to unaltered collagen or gelatin.^87^ Another possible route for controlling fibrillogenesis with inorganic materials is to use particles as nucleators, which has been demonstrated with silicate nanoparticles which underwent sulphation.^55^ These examples from the field of bionanocomposites, centered on the interactions between organic polymers and inorganic colloids, illustrate the advances in recent years, which are reviewed elsewhere.^88^

The biological response of collagen gel networks is not always mediated by cellular response to the fibers. For example, changes in pH have been found to affect subsequent mineralization in gels. By adjusting the pH of acid soluble collagen fibrils to 9, prior to fibrillogenesis, amines along the molecule reacted to produce ammonia. This allowed the collagen fibers to retain electronegative charges that are stable at pH 7, after readjustment.^89^ The reaction only took place before fibrillogenesis and could not be induced after the fiber had formed.^89^ Additional charges along the fiber acted as extra nucleation sites for intramolecular mineralization, leading to increased mineral content both *in vitro* and *in vivo*, in a subcutaneous model.^89^

Tailoring collagen scaffolds requires balancing alterations in the collagen conformation and any possible interactions with the scaffold processing technique. Scaffolds made *via* ice-templating, for example, rely on the use of ice to create porous open structures. The resultant structures depend on the formation and growth of ice, which is, in turn, affected by many of the same factors which modify fibrillogenesis, most notably pH and solutes.^90,91^ Thus, the properties of the ice growth and collagen conformation both impact the final scaffold architecture, and cell response. These sometimes competing effects can be observed when the collagen solution is modified during scaffold production.

Scaffolds can be made from collagen solubilized in both hydrochloric acid (HCl) and acetic acid, but the collagen conformation and subsequent scaffolds is affected by the acid type.^16,91^ Changes in viscosity between collagen solutions hydrated in the two acids suggest that the conformation was altered by the ionic strength of the acids. In HCl, the collagen solution had a higher viscosity, and the resulting scaffolds had a larger pore size. Other studies have also demonstrated a change in the percolation diameter, altering cellular infiltration.^16^ This structure was better able to support the proliferation of fibroblast cells.^91^ While the collagen fibers were affected by the acid type, the decreased scaffold pore size in acetic acid solutions was most likely dominated by a decrease in the ice growth rate by the higher concentration of acetic acid molecules required to achieve the same pH as the HCl solution.

**Fig. 4:**
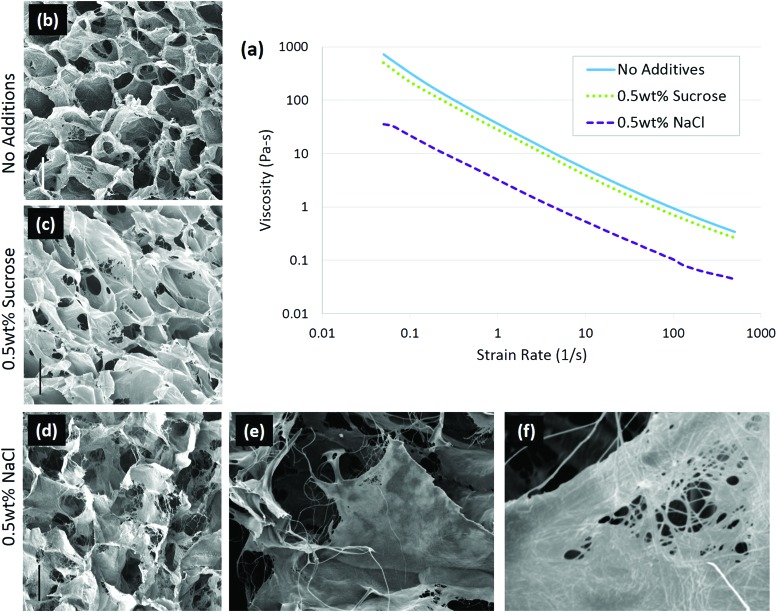
Solute addition to 1 wt% insoluble collagen slurries changed the conformation of the molecule. In ionic solutions, the collagen conformation changed, resulting in a lower viscosity of the slurry (a) than in non-ionic solute. Scaffolds were imaged with (b) no additions, (c) 0.5 wt% sucrose, and (d) 0.5 wt% sodium chloride; scale bar is 100 μm. A highly fibrillar structure is visible with 0.5 wt% NaCl addition at higher magnifications: (e) ×400 and (f) ×3300. Adapted from ref. 92.

In the case of solute addition during scaffold production, it was found that the properties of the solute are key to determining whether the changes in collagen conformation or ice growth play a greater role on the final architecture.^92^ With non-ionic solutes, such as sucrose, changes in the scaffold structure were due to alteration of ice growth. Increasing solutes slow ice growth, leading to scaffolds with a smaller pore size.^93,94^ Ionic solutes, in this case 0.5 wt% sodium chloride (NaCl), affected the collagen conformation more than ice growth. In the presence of NaCl, the collagen became more fibrillar, and the scaffold pore walls had a more fiber-like surface, Fig. 4. The change in conformation is consistent with the ability of NaCl to stabilize collagen triple helices, and was also observed in the decreased viscosity of the collagen slurry, from 36.3 Pa s to 3.2 Pa s, Fig. 4, suggesting the collagen had fewer entanglements.^52^ The conformational change in the collagen was enough to offset the decrease in ice growth rate caused by salt addition, allowing ice crystals to grow significantly greater, resulting in larger pores. In conjunction with structural changes, adhesion of chondrocytes was significantly lowered with the addition of 0.5 wt% NaCl, highlighting the importance of collagen conformation on biological activity.^92^

There is obviously a need to consider the chemical state of the collagen before creating any matrix for biomedical applications. The great potential for tailoring the cellular environment *via* this approach can be applied to collagen gels relatively easily. In the case of scaffolds, it can be difficult to separate the effects of scaffold processing from collagen conformational changes. This area of biomaterials design is not emphasized in current literature, but the obvious biological impact of structural changes makes it an interesting avenue to explore further. However, once a network is formed, it must be stabilized, which requires a different type of collagen modification through the use of cross-linkers.

### 4.2 Modification of chemistry: post network formation

Having discussed the extreme sensitivity of cellular response to both mechanics and chemical stimulus, it is apparent that any processes which alter either property must be carefully considered. The process of stabilizing collagen networks, either gels or scaffolds, *via* cross-linking can affect both properties. On one hand, the covalent cross-linking reactions strengthen the collagen structure to improve handling, slow degradation, and aid cellular mechanosensing. On the other hand, many cross-linking treatments disrupt the cell adhesion ligands necessary for biocompatibility. This dual nature of cross-linking has been demonstrated, with collagen films and fibers, where mechanics and the cross-linking chemistry both influence cellular adhesion.^95,96^

Cross-linkers affect cells by altering the mechanical environment, by changing the number or conformation of ligands present, and possibly by being released as cytotoxic agents during degradation. It is difficult to separate the effects of chemistry from scaffold mechanics. It has been shown that both high substrate elasticity and high ligand affinity increase traction forces exerted by cells and raise the maximum traction force which can be exerted.^84^ As integrin bonding is mediated by mechanosensing and ligand density, biological response to scaffolds is altered.^21^ Thus, collagen scaffold cross-linking can direct biological responses, such as the differentiation of mesenchymal stem cells.^97^ It is important to note that many cross-linkers react at amine and carboxylic acid sites, which are amino acids commonly part of integrin ligands, such as RGD and GFOGER. The removal of these vital groups can alter cellular differentiation, adhesion and migration.^98,99^ It is therefore important to consider the type and amount of cross-linking which is necessary for each particular application.

One of the key considerations when selecting a cross-linking method is to ensure that cytotoxic effects of the cross-linker are minimized. The most well known of the collagen cross-linkers, glutaraldehyde, has been shown in many studies to be effective at stabilizing scaffolds, but it is associated with a high level of cytotoxicity which continues over time due to slow removal of the glutaraldehyde chain during degradation.^100,101^ This severely limits the applications where it can be used, and it has been noted that there is a general lack of cross-linkers for injectable gels which act quickly enough to stabilize the gel without adverse effects.^102^ Other alternatives, such as hexamethylene-diisocynate (HMDIC), also link amide groups, but without the same ability to stabilize collagen networks.^103^ The size and chemistry of the cross-linker can alter the scaffold properties independently of the reaction effectiveness. It was demonstrated on gas foamed scaffolds that cross-linkers with long chains, that are incorporated into the scaffold structure, for example HMDIC, increase water absorption and elastic response.^104^

Not all cross-linkers are incorporated into the scaffold after reacting. During dehydrothermal treatment (DHT), collagen samples are heated to between 100–150 °C under vacuum, to induce carboxylic and amine groups to react. With increasing temperature, the cross-linking density increases. However, temperatures above 150 °C have been shown to lead to denaturation of the collagen, with levels reaching as high as 60% denaturation as the temperature increases to 180 °C.^105^ A water soluble carbodiimide, 1-ethyl-3-(3-dimethyl aminopropyl)carbodiimide hydrochloride (EDC), also catalyzes a reaction between carboxylic acids and amine groups, without being incorporated, lowering potential cytotoxic effects due to the cross-linker itself.^106^

**Fig. 5:**
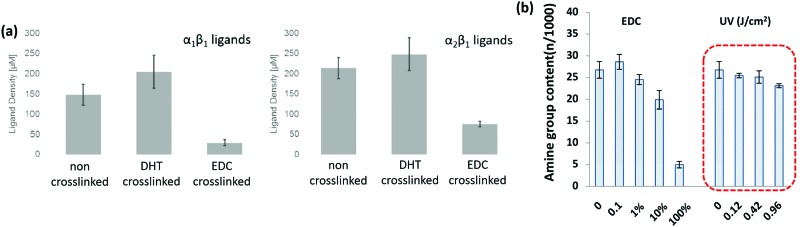
Cross-linking type and amount influences the integrin ligands available for binding. (a) Ligand density of collagen scaffolds after cross-linking with DHT (120 °C, 48 hours), and EDC (14.4 mM EDC). Modified from ref. 107. (b) Amine groups in collagen scaffolds after EDC cross-linking (100% refers to 11.5 mg ml^–1^ EDC) and UV treatment (in J cm^–2^). At high amine content, it is more likely that the GFOGER sequence remains unaltered. Adapted from ref. 108.

A key problem with comparisons between different cross-linking studies is that protocols often differ greatly, which adds variation between reported scaffold strengths. The most common metric for characterizing cross-linking is *via* the overall mechanical strength. However, some evaluation of the total number of cross-links or even the ligand density remaining on the collagen material, might provide a more reliable baseline for comparison.^107^ After stabilization, collagen networks can have a significantly different number of cell adhesion ligands which affects their *in vivo* performance, Fig. 5(a).^107^ Aside from the cross-linking protocol, some of the variation between studies may also be due to different collagens used as starting materials, as this has been shown to have a large effect on integrin ligand density and cross-linking effectiveness.^98^ In order to optimize scaffold chemistry for regenerative medicine, a focus on the underlying principles which drive cellular response would be required. Increasing attention has been given to how cross-linking affects cellular reactions such as adhesion and migration, as these are key to beginning repair during injury.

Preserving the ligands which communicate with cells is extremely important. It has been shown, for example, that cross-linkers which interfere with integrin ligands cause significant decreases in cell attachment, proliferation and migration.^98,109–111^ In general, as the cross-linking strength increases, the cellular response becomes less favorable, even in the absence of cytotoxic effects due to the cross-linker itself. At EDC cross-linking strengths of less than 10% what is normally used in literature, the attachment of HT1080 cells increased by 5 times, and the scaffolds remained stable in physiological conditions.^98^ Cellular proliferation is also affected negatively by cross-linking protocols which maximize mechanical strength.^109,112^ On collagen fibers, increases in mechanical strength, as EDC concentration ranges from 0.25 to 25 mM, significantly decrease cell proliferation.^109^ The same was shown to occur in collagen scaffolds. As EDC concentration was increased from 6 mM to 96 mM, cell proliferation became negligible.^112^ An inverse trend in fibroblast migration with cross-linking strength was demonstrated, as well, when comparing several different cross-linking methodologies.^111^ The cross-linking which yielded the highest mechanical strength showed the least migration of dermal fibroblasts across collagen fibers.^111^

Alternative cross-linking methods which do not rely on the amino acids found in integrin ligands, might be a promising way to optimize scaffold strength while retaining cell compatibility. Efforts to cross-link with UV have shown that ligands and cell attachment are retained, even at the highest cross-linking strengths tested, Fig. 5.^108^ UV cross-linking relies on the reaction of aromatic groups, namely tyrosine and phenylalanine, after the exposure to light in the UV range, around 245 nm.^113^ A trade-off is made between increasing mechanical strength and degradation of the collagen fibers.^101,114^ Enzyme mediated cross-linking is also possible. Transglutaminase, an enzyme similar to LOX, has been shown to alter *D* spacing in collagen fibers, and further to up-regulate integrin subunits β_1_ and β_3_.^115,116^ Copper ions have also been found to function as a cross-linker on collagen fibrils. After reacting copper with the collagen, the network had enhanced protease resistance without changing the structure.^117^ Another tack is to use self cross-linking systems.^18,118^ In the case of collagen and chitosan, blends can achieve the same stability through internal chemical reactions as they can through the use of chemical cross-linkers.^18^ Finally, cross-linking *via* peptides is another route, allowing different functionality to be built in, including ligands for collagenase (MMP sequences). This strategy has been shown to be able to control migration of cells independently of the total number of cross-linkers.^119^ However, as an emerging field of tailored cross-linking, there remains many avenues of exploration for peptide cross-linkers.^120^ All of the methods above react in ways which do not eliminate vital ligands for cell culture and proliferation, while still creating mechanically stable scaffolds.

**Fig. 6:**
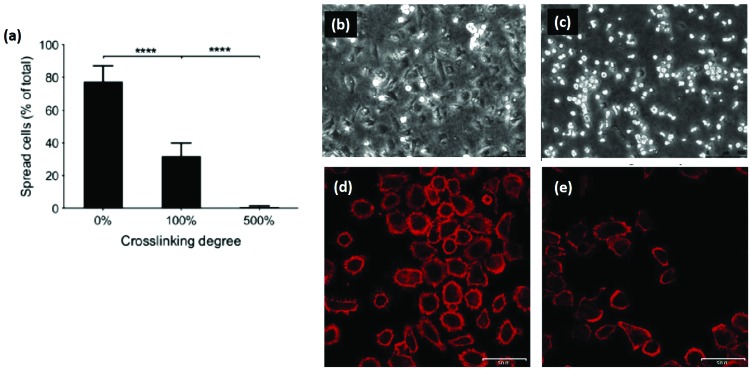
Cross-linking can remove cell adhesive ligands on the collagen substrate. (a) Increased EDC cross-linking of collagen films reduced the spreading of HT1080 cells. (100% crosslinking refers to 1.15 g EDC and 0.276 g NHS per gram collagen.) (b and c) Phase contrast images of (b) cells on uncross-linked films and (c) 500% cross-linked films. In comparison, after reintroducing GFOGER peptides to 500% cross-linked films, cell adhesion was restored: (d) 500% cross-linking with peptides, (e) 500% cross-linking without peptides. Scale bar (d and e) is 50 μm. Modified from ref. 121.

Another promising area of research is reintroducing ligand sequences into cross-linked collagen structures. It has been demonstrated that peptides, containing sequences for integrin binding, can up-regulate cell binding and adhesion, especially if they have a native conformation, like cyclic RGD.^80^ By reintroducing the integrin ligand GFOGER into an EDC cross-linked scaffold, a recent study showed significant improvement in cell attachment and spreading for several cell types, Fig. 6.^121^ By tailoring the peptide attachment site, the addition could be localized and controlled as a further means of tailoring the structure.^121^ This might be a major advance to overcome the negative affects inherent in many cross-linking processes.

Despite improvements in mechanical properties with cross-linking, collagen materials remain very soft networks. With the competing forces of stability and cell response, it would be well worth considering individual applications for collagen scaffolds, to determine the properties which are most needed. If a small gain in mechanical strength does not greatly improve the function of the construct, then it might be best to decrease the cross-linking to optimize cellular signalling instead. However, new methods of stabilizing the collagen networks are emerging, such as ions or self cross-linking polymer systems, which have yet to be fully explored. Also, the possibility of reintroducing integrin ligands into the collagen network, may finally overcome the conflict between mechanics and chemistry.

## 5. Conclusions

Collagen networks are one of the cornerstones of regenerative medicine. Building collagen networks relies on a multi-step process of fibrillogenesis, which is sensitive to the properties of its environment. The most promising ways to alter collagen structure during fibrillogenesis include: changing the pH, the ionic strength of the solution, or the amino acid sequence of the collagen molecule itself. Biological activity of collagen is mediated through integrin receptors, which interact with the high affinity ligand, GFOGER, on the helical collagen fiber. During scaffold production, collagen chemistry can be altered either before the network is formed, or after scaffold formation. Regardless of which stage collagen chemistry is altered, the biological response is affected. A key point emerging from literature is the importance of preserving ligand sites along the collagen chain to ensure that biocompatibility is not lost during scaffold production. The ability to reintroduce integrin ligands, into a cross-linked structure, has recently opened up new avenues for controlling cell behavior on collagen networks. Consideration of the collagen structure and chemistry, inherent to collagen scaffolds, is a promising area for further exploration.
